# Bisphosphonate Related Osteonecrosis of the Jaw: A Study of 18 Cases Associated with Fungal Infection

**DOI:** 10.1155/2014/869067

**Published:** 2014-02-18

**Authors:** V. Aftimos, T. Zeinoun, R. Bou Tayeh, G. Aftimos

**Affiliations:** ^1^National Institute of Pathology, Faculty of Medicine, Lebanese University, Rafic Hariri Campus, Baabda, Hadath, Lebanon; ^2^Department of Oral and Maxillo-Facial Surgery, Faculty of Dentistry, Lebanese University, Rafic Hariri Campus, P.O. Box 6573/14, Museum, Badaro, Beirut, Lebanon

## Abstract

Osteonecrosis of the jaw (ONJ) is a serious complication associated with oral and intravenous bisphosphonate therapy. Its pathogenesis is not well understood and its management is difficult. Microbiological investigations have detected a variety of oral pathogens such as *Actinomyces, Enterococcus, Candida albicans*, *Aspergillus*, *Haemophilus influenzae,* alpha-hemolytic streptococci, *Lactobacillus, Enterobacter*, and *Klebsiella pneumoniae*. To better treat it, it is important to understand its causes and complications. *Materials and Methods*. Our present study addresses a microscopic observation of curetted jaw necrotic lesions related to bisphosphonates. *Results*. A mycotic infestation has been found in all of the 18 cases studied. *Discussion*. An identification of the fungal agent and its incrimination in the pathogenesis of bisphosphonates related osteonecrosis of the jaw could change radically the management of this condition.

## 1. Introduction

With the increased use of bisphosphonates in the treatment of osteoporosis and other conditions as multiple myeloma and [1] bone metastasis and their complications (hypercalcemia, pain, pathologic fractures, nephrotoxicity, electrolyte abnormalities, and spinal cord compression) [1], a side effect difficult to manage was observed: bisphosphonates related osteonecrosis of the jaw (BRONJ). BRONJ was described for the first time in the literature in 2003 [2–5].

“The American Society for Bone and Mineral Research” defines BRONJ as an area of exposed bone in the maxillofacial region that does not heal within 8 weeks after its identification, by a health care provider, in a patient who is receiving or has previously been receiving BP and who has not had radiation therapy to the craniofacial region) as the exposure of bone in the maxillofacial area for more than 8 weeks. The patient should have been treated by bisphosphonates and should not have a history of radiotherapy to the jaw [6]. Osteonecrosis of the jaw can also be triggered by factors other than the administering of BPs. It occurs after radiation therapy of the facial area, trauma (osteotomy of the jaw bone or during intubation), viral infection (herpes zoster or HIV), fungal infection with *Aspergillus* [7], circulatory insufficiency, local application of chemical agents in dental treatment, inhaling cocaine, and osteomyelitis [8]. So bisphosphonates may not be the primary cause. Also in a recent study, Naik and Russo observed the presence of *Actinomyces* as an underrecognized agent in pathogenesis, and timely actinomycosis-specific treatment may improve outcome [9]. The pathogenesis behind this osteonecrosis has not been elucidated yet but many etiological factors have been incriminated [4]. Also the lack of science-based guidelines for the management of patients with this disease makes treatment empiric [3]. In the cases that we are going to describe, our goal is to demonstrate a correlation between a fungal infection and BRONJ: is it a cause or a consequence?

## 2. Materials and Methods

The population studied was recruited from the entire Lebanese territory in a private laboratory of anatomo-pathology “National Institute of Pathology.” The cases of osteonecrosis between January 2008 and June 2013 were reviewed. 31 cases of osteonecrosis of the jaw were identified during this period. 19 cases were selected, as the patients were known to be treated by bisphosphonates (I.V). One case was excluded because the patient was also treated by radiotherapy. Four cases were reevaluated because of missing data, the evaluation for a fungal infection was not made initially.

**Figure 1:**
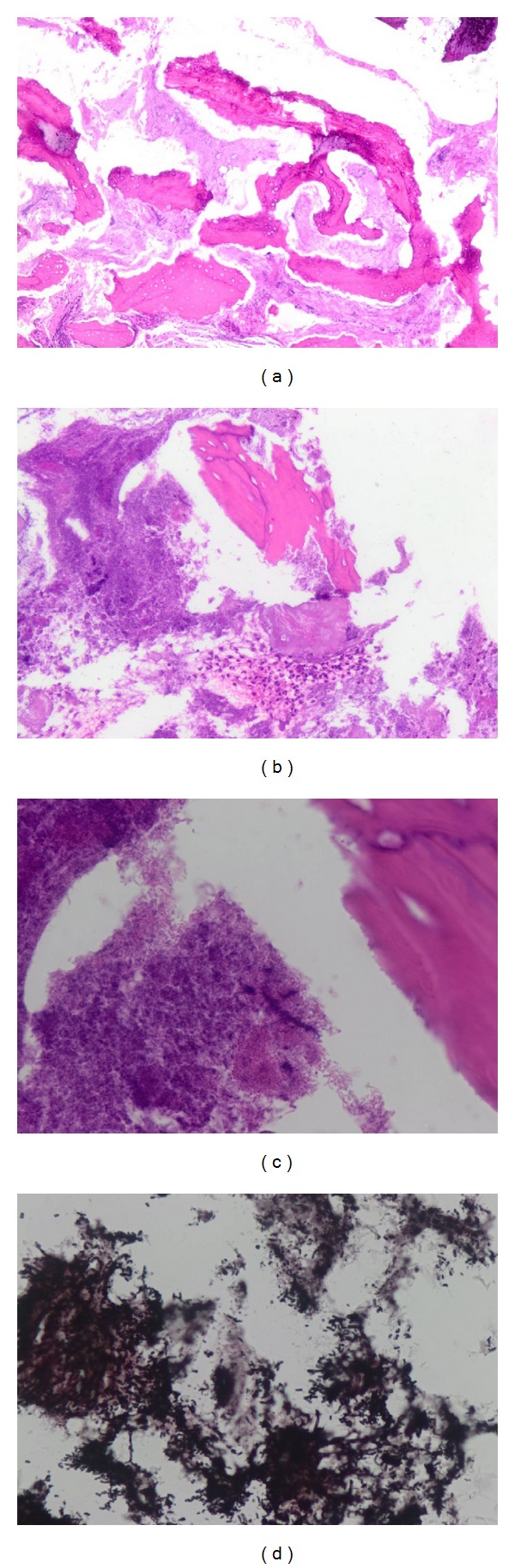
(a) H&E ×40. Overview: necrotic bone medullary spaces filled with hyphae. (b) H&E ×100 necrotic bone, hyphae, and altered Polymorphs. (c) H&E ×400. Detail: hyphae, and spores in medullary spaces. (d) Groccott ×400. Hyphae and spores.

All cases studied are curetting of lesions of osteonecrosis identified by the treating physician. The specimens are fixed in formalin at 10%. They are then decalcified by nitric acid at 10% and embedded in paraffin. Slides are cut at 5 microns and stained by H&E and PAS (Figures 1(a), 1(b), and 1(c)). Some cases were also stained by Grocott (Figure 1(d)).

The slides were then examined on an optical microscope.

## 3. Results

18 cases of BRONJ were reported. The population consists of 3 men (17%) and 15 women (83%). The mean age was 58.3 with a minimum of 39 and maximum of 82. A mycotic infestation was found in the 18 cases (100%), but the nature of the fungal agent was not determined. Five out of 18 cases were treated by zoledronic acid (Zometa 4 mg/100 mL per IV). The rest of the patients were treated by other I.V bisphosphonates. One patient was treated for Histiocytosis X, another for colon cancer, and a third one for breast cancer. Data is missing concerning the underlying disease for the other 15 patients.

On histologic sections, Figures 1(a), 1(b), and 1(c) we mainly noticed necrotic bone trabeculae and leucocytes, (especially alterated neutrophils) infiltrating medullary spaces. Mycotic spores and hyphae were also noted and were positive with PAS and Grocott stains (Figure 1(d)).

## 4. Discussion

Bisphosphonates are pharmacologic compounds characterized by high tropism to bone tissue. They affect bone metabolism by inhibition of osteoclast recruitment, proliferation, differentiation and function, resulting in avascular necrosis [10–12]. They inhibit the osteoclastic activity and thus the bone remodeling which is an important component of repair [11]. In vitro, they have a direct toxic effect on the soft tissue of the oral cavity but we still do not know if their concentration in vivo is enough to cause the same effect. Bisphosphonates also diminish neoangiogenesis [13, 14]. Zoledronic acid has marked antiangiogenic properties that could augment its efficacy in the treatment of malignant bone disease and extend its potential clinical use to other diseases with an angiogenic component [15].

In a recent study by Wehrhan et al. [16], mucoperiosteal tissue samples from BRONJ cases and controls were assessed for vascularization with CD31 staining and neoangiogenesis by CD105 evaluation. It was reported that although there was no difference in vascularization between sample groups, there were significantly fewer CD105-positive vessels in BRONJ samples suggesting that neoangiogenesis was suppressed in BRONJ cases [15, 17, 18]. However, angiogenesis is an essential factor in healing of wounds. Also, normal vascularization represents an essential requirement for tissue homeostasis. Also, local immunity, and regeneration capacity are important pre-requirements for repair of all vital tissues of the body, especially in case of bone tissue that displays a high turn over rate. CBCT examinations of patients taking bisphosphonates might be able to show early bone alterations associated with the treatment [19]. New studies are incriminating a new agent in the pathogenesis of osteonecrosis: the presence of *Actinomyces* or *Actinomyces*-like organisms was demonstrated almost constantly in histological studies [19–28]. Other studies have shown the presence of *Fusobacterium*, *Eikenella*, *Bacillus*, *Staphylococcus*, and *Streptococcus* as well as *Actinomyces* [21, 22, 29, 30]. If they are part of the biofilm or they are invasive agents is not yet determined.

The most advanced studies in this field concern *Actinomyces*. The presumed sequence is the following: bisphosphonates weaken host defenses in the oral cavity and establish a niche in the bone. An important but not universal condition to infection is the disruption of the mucosa by dental surgery, trauma, bad oral hygiene, ill-fitted dentures, and so forth [23, 25, 27, 31]. Bisphosphonates inhibit the replication cycle of keratinocytes and thus play a role in the disruption of the mucosa and the delay in repair [22]. The environment is at that moment ideal for the development of actinomycosis. Because viable bone can be found in specimens infected by *Actinomyces*; some authors think that *Actinomyces* infects the viable bone and does not colonize it secondarily after necrosis [22, 31]. Others think that because *Actinomyces* is a commensal bacteria of the oral cavity, its presence in necrotic bone must be secondary and is not an etiological factor [27, 30, 32].

**Figure 2:**
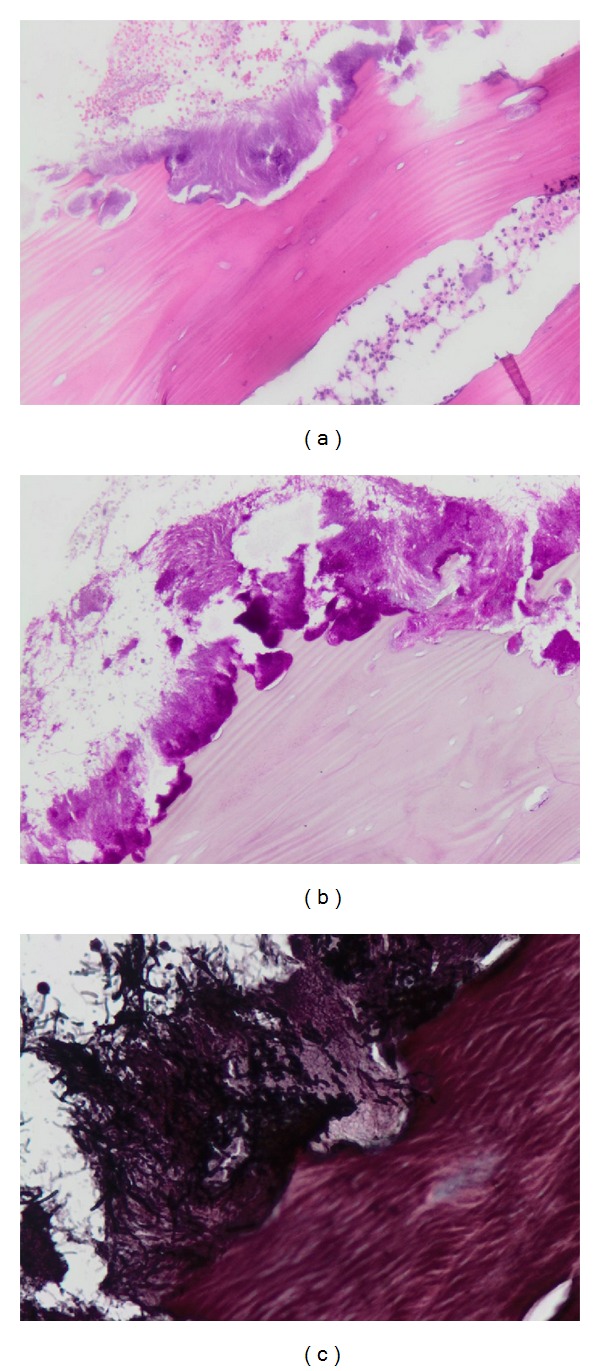
(a) H&E ×100. Necrotic bone trabecula with hyphae and altered polymorphs. (b) PAS ×100 necrotic bone with hyphae. (c) Grocott ×400 hyphae and spores.

In our study, unlike all others, our incriminated agent is a fungus [32] and not a bacteria—it is stained by PAS and Grocott (Figures 2(a), 2(b), and 2(c)). However, it is important to stress that cultures taken from an exposed jaw bone may give misleading results because the isolates may include nonpathogenic microorganisms that are colonizing the site [33]. We already know that when an *Actinomyces* is associated, its specific treatment improves significantly the prognosis of BRONJ [9] and as we established previously, 100% of our BRONJ were associated with a fungal infection.

The BRONJ therapy remains an unresolved problem and there are no evidence-based guidelines. On the basis of the recent literature it is necessary to consider the treatment of patients affected by early BRONJ stages with combined conservative surgical strategies to obtain a greater control of these lesions for longer periods of time. The previous considerations support the hypothesis that the medical therapy and alternative noninvasive therapies (LLLT, OZ OTI) can be effective in jawbone and mucosa defects connected to BRONJ development [26].

And so, within the same way of thinking, by determining the nature of this agent as well as the sequence, cause or secondary infection, a specific antifungal treatment could be added to the management of BRONJ and it could radically change its course. A wider analysis of the fungal infection of BRONJ sites is mandatory to clarify its role.
